# Rapid and Direct Transport of Cell Surface APP to the Lysosome defines a novel selective pathway

**DOI:** 10.1186/1756-6606-3-11

**Published:** 2010-04-21

**Authors:** Angela Lorenzen, Jonathan Samosh, Kenneth Vandewark, Pieter H Anborgh, Claudia Seah, Ana C Magalhaes, Sean P Cregan, Stephen SG Ferguson, Stephen H Pasternak

**Affiliations:** 1 J. Allyn Taylor Centre for Cell Biology, Molecular Brain Research Group, Robarts Research Institute, Schulich School of Medicine, the University of Western Ontario, London, Ontario, N6A 5K8, Canada; 2 Departments of Clinical Neurological Sciences, Schulich School of Medicine, the University of Western Ontario, London, Ontario, N6A 5K8, Canada; 3 Department of Physiology and Pharmacology, Schulich School of Medicine, the University of Western Ontario, London, Ontario, N6A 5K8, Canada

## Abstract

**Background:**

A central feature of Alzheimer's disease is the cleavage of the amyloid precursor protein (APP) to form beta-amyloid peptide (Aβ) by the β-secretase and γ-secretase enzymes. Although this has been shown to occur after endocytosis of APP from the cell surface, the exact compartments of APP processing are not well defined. We have previously demonstrated that APP and γ-secretase proteins and activity are highly enriched in purified rat liver lysosomes. In order to examine the lysosomal distribution and trafficking of APP in cultured cells, we generated constructs containing APP fused to a C-terminal fluorescent protein tag and N-terminal HA-epitope tag. These were co-transfected with a panel of fluorescent-protein tagged compartment markers.

**Results:**

Here we demonstrate using laser-scanning confocal microscopy that although APP is present throughout the endosomal/lysosomal system in transfected Cos7 and neuronal SN56 cell lines as well as in immunostained cultured mouse neurons, it is enriched in the lysosome. We also show that the Swedish and London mutations reduce the amount of APP in the lysosome. Surprisingly, in addition to its expected trafficking from the cell surface to the early and then late endosomes, we find that cell-surface labelled APP is transported rapidly and directly from the cell surface to lysosomes in both Cos7 and SN56 cells. This rapid transit to the lysosome is blocked by the presence of either the London or Swedish mutations.

**Conclusions:**

These results demonstrate the presence of a novel, rapid and specific transport pathway from the cell surface to the lysosomes. This suggests that regulation of lysosomal traffic could regulate APP processing and that the lysosome could play a central role in the pathophysiology of Alzheimer's disease.

## Background

One of the pathological hallmarks of Alzheimer's disease (AD) is the production and cerebral deposition of the β-amyloid (Aβ) peptides. Aβ peptides are generated by the sequential proteolysis of the Amyloid Precursor Protein (APP). β-Secretase (BACE) performs the first cleavage of APP at an extracellular/luminal 'β-site' which removes the bulky extracellular domain of APP [1,2]. This initial cleavage is followed by a second cleavage at a 'γ-site' within the transmembrane domain of APP by γ-secretase to yield the 40-42 amino acid Aβ peptide [3,4].

APP is a type 1 transmembrane protein that is transported to the cell surface where it undergoes rapid endocytosis based upon a C-terminal tyrosine-based sorting signal. APP then either recycles back to the cell surface or is targeted to late endosomes/lysosomes [5-10]. Many lines of evidence suggest that APP processing by secretases occurs in the endosomal/lysosomal system (reviewed in [11]). Aβ production is reduced by blocking the internalization of cell surface APP [12,13], and blocking the acidification of the endosomal-lysosomal system [8,14,15]. Furthermore, amyloidogenic APP fragments accumulate in lysosomes after treatment with protease inhibitors and in presenilin-1 knockout cells lacking γ-secretase activity [15-18]. However, there is also evidence suggesting that APP processing may occur in other compartments and the site of these critical biochemical events remains controversial [18-21].

The Swedish mutation causes early onset Familial AD by increasing the rate of β-cleavage by 5-10 fold [22] and is proposed to alter the trafficking of APP. This mutation partly disrupts polarized sorting of secreted APP [23] and appears to undergo β-cleavage during transit to the cell surface [24,25]. In contrast, the "London" mutant involves a missense mutations at codon 717 and increases the relative amount of the more toxic Áβ42 produced by γ-cleavage [26], but is not proposed to alter trafficking.

Previously, we provided evidence that the lysosome might be an important site of Aβ production [27,28]. In the present study, we demonstrate that APP is enriched in the lysosome compared to early and late endosomes in transfected cultured cells and immunostained mouse neurons and that cell surface APP is transported rapidly and directly to the lysosomal compartment. This rapid lysosomal transport is blocked by the presence of the Swedish and London mutations. Taken together, our experiments demonstrate a novel highly selective rapid and direct sorting pathway from the cell surface to the lysosome.

## Results

### Localization of FL-APP and βAPP in the endosomal/lysosomal compartment

**Figure 1:**
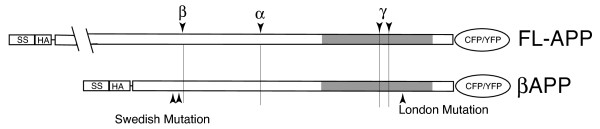
**Overview of Constructs**. Shaded region denotes the transmembrane domain. Beta-, alpha- and gamma symbols denote secretase cleavage sites. Swedish and London mutations are shown. SS - signal sequence; HA- HA epitope tag.

**Figure 2:**
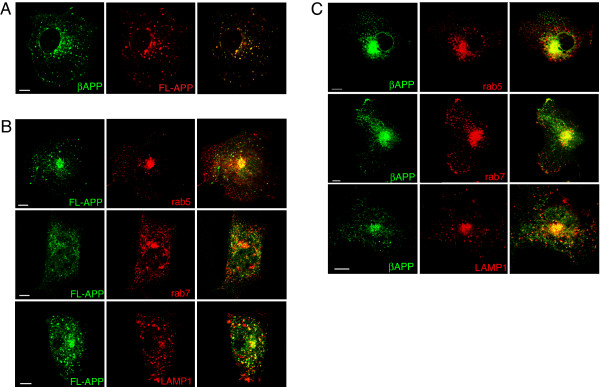
**APP is preferential expressed in the lysosome in Cos7 cells**. Cos7 cells were transiently co-transfected with the fluorescent -tagged APP and compartment marker proteins and imaged using laser scanning confocal microscopy. **A**. Cos7 cells were transiently co-transfected with βAPP-CFP (green) and the full-length APP-YFP (FL-APP) (red). **B**. Cos7 cells transiently co-transfected with βAPP-CFP or YFP (green) constructs along with fluorescent-tagged compartment markers (red) **C**. Cos7 cells were transiently co-transfected with βAPP-CFP (green) a constructs along with compartment markers as indicated (red). Scale Bar = 10 microns.

We first examined the distribution of fluorescent protein tagged APP in non-neuronal cells to determine if would indeed be enriched in lysosomes and other endocytic compartments. To eliminate the possibility of interference from large (likely non-secretase related) cleavage products of APP that we and others have observed in purified lysosomes (data not shown) and poorly defined sorting signals in the luminal domain of APP [23,25,29], we generated a shorter APP constructs beginning 12 amino acids upstream of the β-cleavage site, referred to as βAPP (Figure 1). We then co-transfected Cos7 cells with either full-length APP-YFP or -CFP (FL-APP) or β APP-CFP along with compartment markers that label early endosomes (Rab5), late endosomes/lysosomes (Rab7) and lysosomes (LAMP1). We found that FL-APP and βAPP were colocalized to the same intracellular compartments in transfected Cos7 cells (Figure 2A). Moreover, both APP constructs exhibited extensive colocalization with Rab5, Rab7 and Lamp1 (Figure 2B and 2C). Thus the truncation of the N-terminus of APP did not appear to alter the intracellular trafficking of βAPP.

### APP is present in the endosomal/lysosome system in neural cells and neurons

To examine APP distribution in neural cells, we adopted the SN56 cell line. These cells are a hybrid cell line generated by fusing dissociated embryonic mouse septal neurons with N18TG2 neuroblastoma cells. SN56 cells possess neuronal morphology and cholinergic phenotype when differentiated and express APP [30-32].

In order to compare the levels of APP within various compartments of the endosomal/lysosomal system, we set out to quantitate the relative amounts of APP colocalized with organellar compartment markers. Colocalization analysis requires the setting of brightness intensity threshold to determine the level of intensity that is considered 'positive' for each channel and separating it from 'background' expression. This can then be quantified by counting the pixels (or the percentage of pixels) that are positive for both fluorescent labels. The process of thresholding images is inherently problematic because it requires the arbitrary determination of the level of expression is considered significant or positive. Furthermore the brightness of signals in fluorescence images is highly dependant on numerous variables that are difficult to control such as variations in cell-to-cell protein expression, fluorophore brightness, and image acquisition. To circumvent these problems, we adopted a strategy described by Hutcheon *et al *[33] (also discussed in [34,35]), which sets thresholds based on a fixed percentage of the brightest pixels in an image. This allows for the identification of positive pixels that is unbiased (it does not require the judgment of the observer on an image to image basis) and is relatively unaffected by parameters of image acquisition or the level of protein expression. This strategy assumes that the most important site of a protein will have the largest amount of the protein and therefore brightest signal. This strategy also improves sub-cellular organelle identification, as proteins that are considered to be markers of distinct compartments are often present at lower levels in other compartments. In this study, we set thresholds to identify the brightest 2% of pixels in each image. This level was determined empirically to consistently identify APP in punctuate organelle-like patterns.

**Figure 3:**
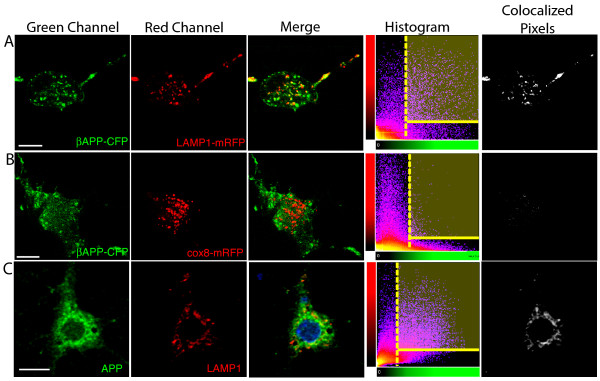
**Quantitation of APP enrichment in transfected cells and primary neurons**. SN56 cells (A-B) were transfected with transfected with APP-CFP (green channel) and either LAMP1-mRFP or Cox8-mRFP (red channel) and imaged by confocal microscopy. Mouse neurons (C) were immunstained with Anti-C-terminal APP (green channel) and Anti-LAMP1 (red channel). The co-localization of the green and red channels can be view qualitatively in the yellow (merge) images and the histograms of these images are shown. To quantify colocalization, the brightest 2% of green (to the right of the dotted yellow line) and red pixels (above the yellow solid line on the histograms) is selected. The colocalized pixels are indicated in white in the Colocalized Pixels panels.

An example of this analysis is shown in Figure 3 which demonstrates the colocalization of APP and LAMP1 in SN56 cells (Figure 3A-B) transfected with βAPP-CFP (green) and LAMP1 or Cox8 compartment markers tagged with mRFP (red), or mouse neurons immunostained with APP (green) and LAMP1 (red) antibodies (Figure 3C). The merged green-red image shows clear colocalization of APP and LAMP1 (but not Cox8) in yellow, and histograms of the intensities of the green and red channel are shown. The brightest 2% of pixels in the green channel was selected as a threshold intensity (the region on the histogram to the right of the yellow dotted line) that also fall within the brightest 2% of red pixels (the bound box in the upper right corner of the histogram). These colocalized pixels are identified in a colocalization channel (white) and the percentage of the selected green pixels that are also red is determined as the percent colocalized. Note that the absolute intensity of the brightness threshold varies slightly from image to image, although the portion of the pixels remains the same. In this figure 49% of threshholded βAPP-CFP is colocalized with LAMP1, whereas only 4% of βAPP-CFP is colocalized with Cox8 (Figure 3A and 3B). Similarly, 54% of APP is colocalized with LAMP1 in the selected immunostained mouse neuron (Figure 3C).

**Figure 4:**
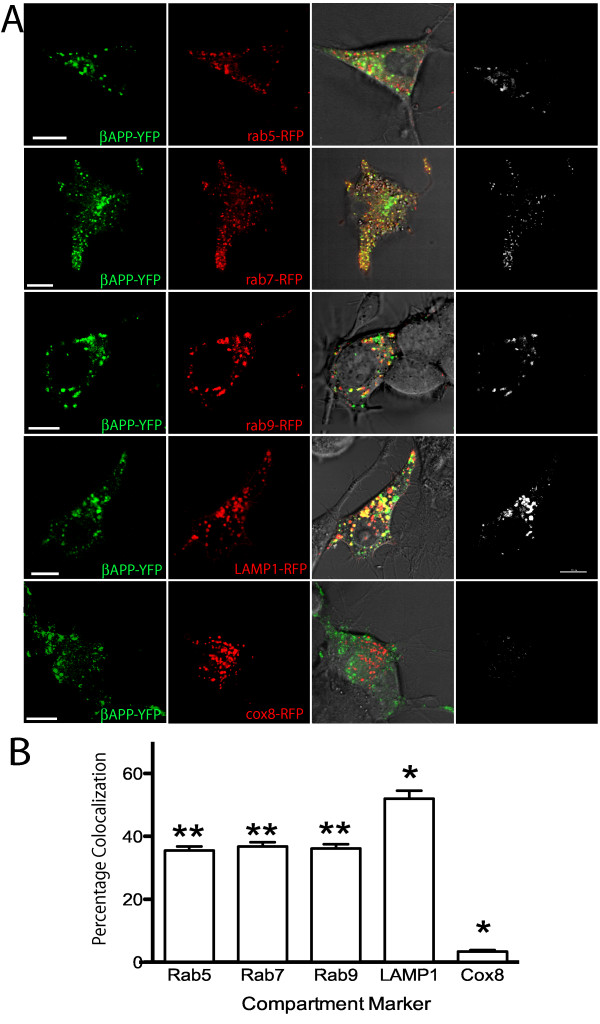
**APP is Enriched in the Lysosome in Neuronal SN56 cells**. SN56 cells were transiently co-transfected with fluorescent-tagged APP and compartment marker proteins and imaged using laser scanning confocal microscopy. **A**. SN56 cells were transiently co-transfected with the βAPP-CFP (shown in green) constructs along with compartment markers (red) as indicated demonstrating preferential co-localization of βAPP with lysosomal marker LAMP1. Colocalized pixels were identified as in figure 3 and displayed in the white colocalization channel. Scale Bar = 10 microns. **B**. The colocalization of the brightest 2% of pixels of APP and compartment markers was quantitated by Imaris software. Values are expressed as the mean ± SEM for a minimum of 50 cells each for Rab5, Rab7, Rab9 and LAMP1, and 20 cells for Cox8, drawn from at least 4 independent transfections. * indicates statistically significant difference from all other compartment markers (p < 0.05). ** indicates compartment markers that are statistically different from LAMP1 and Cox8, but not different from each other each other (p < 0.05).

Examining APP colocalization with endosomal/lysosomal compartment markers in transfected SN56 cells (Figure 4), we find that subcellular distribution of βAPP-CFP in neuronal SN56 cells was similar to that found in Cos7 cells. We found that βAPP was extensively colocalized with markers of early endosomes (Rab5), late endosome/lysosomes (Rab7), late endosomes (Rab9), and lysosomes (LAMP1) but not mitochondria (Cox8) (Figure 4A). Colocalization analysis was preformed (as described above) to generate images showing the colocalization of the brightest 2% of pixels (shown in white). Quantification of the brightest 2% of pixels revealed that the extent of colocalization of intracellular protein βAPP with Rab5, Rab7, Rab9 and LAMP1 was 35 ± 1.4%, 37 ± 1.4%, 36 ± 1.4%, and 52 ± 3%, respectively (Figure 4B). A 3D image stack of APP colocalization with LAMP1 in an SN56 cell is shown in Additional file 1, Supplemental movie S1. The relatively high levels of colocalization of βAPP observed in each compartment likely reflects the expected transit of APP through these compartments. Cox8, a mitochondrial marker, exhibits essentially no colocalization (3.4 ± 0.4%) with βAPP. Moreover, βAPP was significantly enriched in the lysosome compared to the other intracellular compartments (p < 0.05).

**Figure 5:**
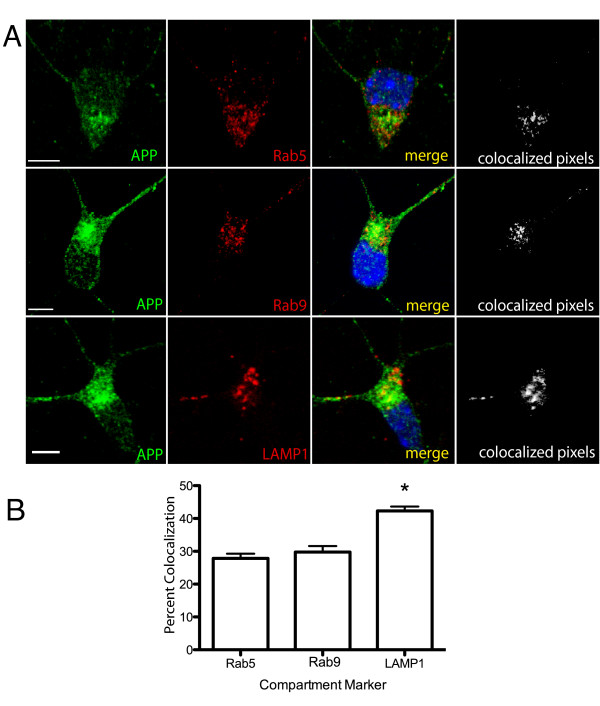
**APP is enriched in the lysosome in cultured mouse neurons**. **A**. Cultured mouse neurons were fixed and immunostained with antibodies against APP (green) or compartment markers (red) and nuclei were counterstained with Hoechst dye (blue). Images were acquired using laser scanning confocal microscopy demonstrating the green, red and merged image channels. An additional channel was generated showing the where the brightest 2% of red and green pixels are colocalized (See Figure 3). Scale bar = 5 microns. **B**. Quantitation of colocalization of the brightest 2% of pixels of APP and compartment makers. Each bar represents with mean ± SEM of at least 3 independent immunostaining experiments, imaging total of at least 25 cells. * indicates the LAMP1 staining is significantly different from Rab5 or Rab9 (P < 0.05).

To confirm that this colocalization was not due to overexpression of APP, we examined the distribution of endogenously expressed APP in cultured mouse primary cortical neurons immunostained with antibodies that recognize the C-terminal of APP along with Rab5, Rab9 and LAMP1. Similar to our findings in SN56 cells, endogenous APP was extensively colocalized with markers of the early endosomal (Rab5), late endosomal (Rab9) and lysosomal (LAMP1) compartments (Figure 5). Quantification of images revealed that the extent of colocalization of endogenous APP protein with Rab5, Rab9 and LAMP1 was 28 ± 1.3%, 31 ± 1.9%, and 42 ± 1.3% respectively (Figure 5B). A 3D image stack of APP colocalization with LAMP1 in a mouse neuron is shown in Additional file 2, Supplemental movie S2. As with the transfected cells, endogenous APP was significantly enriched in the lysosomal compartment (p < 0.05) indicating that the fluorescently-protein tagged proteins expressed in SN56 cells exhibited a subcellular localization pattern that mirrors that of the endogenous protein.

### Effect of Swedish and London mutations on the subcellular localization of APP

**Figure 6:**
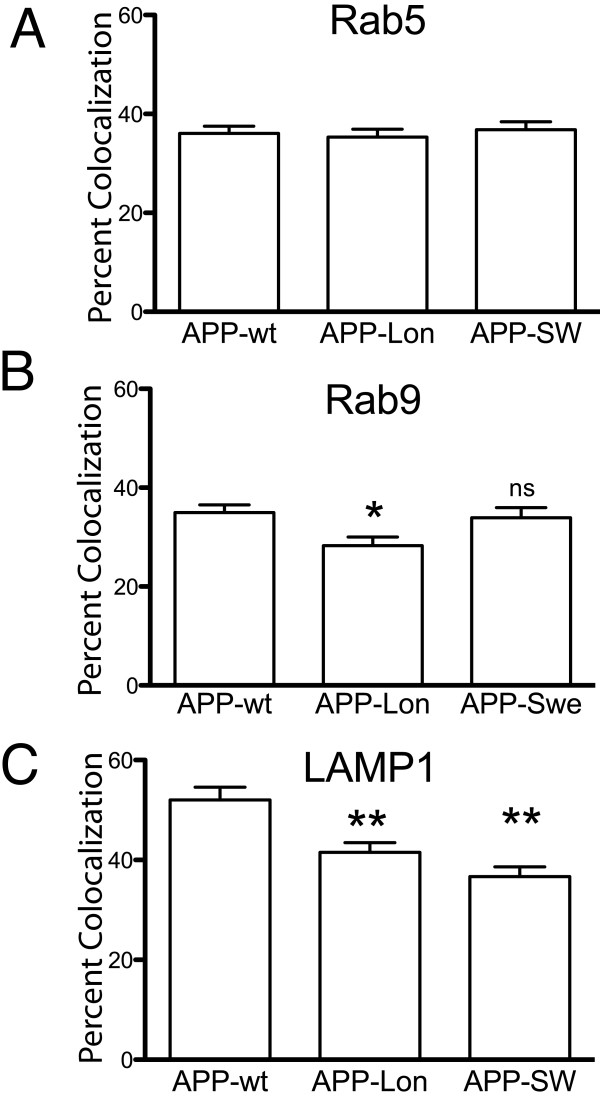
**London and Swedish Mutations reduce APP levels in late endosomal and lysosomal compartments**. SN56 cells were transiently co-transfected with the βAPP-CFP construct with or without the London or the Swedish mutant along with compartment markers. Representative fixed cells were imaged using laser-scanning confocal microscopy. The colocalization of the brightest 2% of pixels of APP mutants with Rab5 (A), Rab9 (B) and LAMP1 (C) was quantitated using Imaris software. Values are expressed as the mean ± SEM for a minimum of 50 cells drawn from at least 4 independent transfections. * indicates statistical difference from wild type and all other compartment markers (p < 0.05). ** indicates compartment markers that are statistically different from wild type (p < 0.05) but not from each other. Scale bar = 10 microns.

Since both the Swedish and London mutations were previously demonstrated to alter Aβ production and might be associated with altered APP trafficking [14,25], we introduced each of these mutations into separate βAPP-CFP construct and examined the steady state colocalization of the resulting mutants with Rab5, Rab9 and LAMP1. We found that neither the London nor the Swedish mutation altered the relative colocalization of βAPP with Rab5 when compared to wild-type βAPP (Figure 6A). However, we found that the London mutation reduced the extent of βAPP localization to both the late endosomal (Rab9) and lysosomal (LAMP1) compartments (Figure 6B and 6C). In contrast, while the Swedish mutation did not affect the extent of βAPP colocalization with Rab9, it significantly reduced βAPP localized to the lysosome.

### Cell surface βAPP internalization to lysosomes

**Figure 7:**
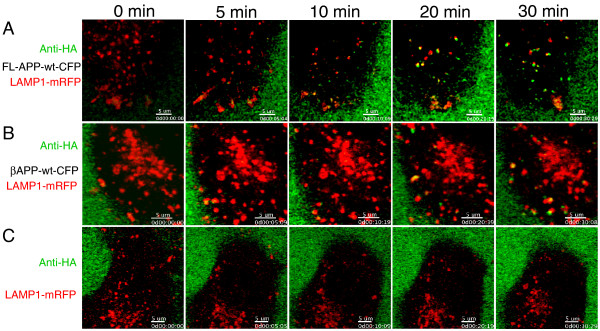
**APP is rapidly internalized to the lysosome in live Cos7 cells**. Cos7 cells were transiently co-transfected with wild type full length FL-APP-CFP (A) or βAPP-CFP (B) constructs along with the LAMP1-mRFP compartment marker construct. Individual cells were selected and fluorescently-labeled anti-HA antibody was added to the media. Laser confocal images were then acquired at approximately 2 frames/min.

The high levels of APP in the lysosome could be the result of rapid trafficked to this compartment. Therefore, we examined the trafficking of wild-type βAPP to LAMP-1 positive lysosomes by imaging live Cos7 cells transfected with LAMP1-mRFP with and without either HA-tagged APP (HA-APP-wt-CFP) or βAPP (HA-βAPP-wt-CFP). Co-transfection of cells was confirmed by observing the fluorescent protein tags. Subsequently, AlexaFluor 647 conjugated HA antibody was added to confocal dishes and the internalization of cell surface antibody-labeled APP protein was imaged. We expected to observe only low levels of APP and βAPP with LAMP1 within 15-30 min following the labeling of cell surface APP protein with the fluorescent antibody. However, we found that both wild-type FL-APP and βAPP exhibited extensive colocalization at early time points (5-10 min) in LAMP1 positive vesicular structures that were found in close juxtaposition with the plasma membrane (Figure 7A and 7B, Additional file 3, Supplemental movie S3 and Additional file 4, Supplemental movie S4). However, the uptake of AlexaFluor-labeled HA antibody was not non-specific as no uptake was observed in cells lacking HA-tagged APP constructs but containing LAMP1-mRFP alone (Figure 7C). The results suggested that APP was rapidly localizing to lysosomes immediately following endocytosis.

### APP transport to the lysosome is both rapid and selective

**Figure 8:**
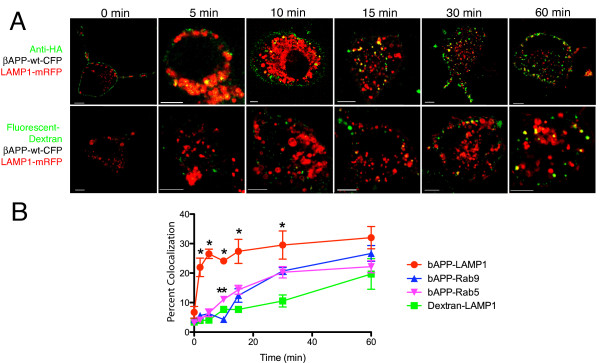
**APP internalization into the endosomal/lysosomal system in SN56 cells**. **A**. Representative images of the uptake of anti-HA antibody or fluorescent dextran (green) to lysosomes labeled with LAMP1-mRFP (red). **B**. Internalization of surface labeled APP was quantitated by determining the co-localization of the brightest 2% of pixels with fluorescent-tagged compartment marker proteins. Each point represents the Mean ± SEM for at least 3 independent experiments examining 4 representative cells each. These means were compared statistically using and ANOVA at each time point. * indicates statistical significance between LAMP1 and all other markers (P < 0.05). ** indicates Rab5 is statistically difference from Rab9 (P < 0.05).

To further examine the lysosomal transport of cell surface APP protein, we established the time course of colocalization of surfaced-labeled βAPP-wt-CFP with early endosomes (Rab5), late endosomes (Rab9) and lysosomes (LAMP1) in SN56 cells. As a control, the non-specific uptake of dextran to these compartments was also examined. Figure 8A shows representative images of the internalization of AlexFluor 647 anti-HA antibody and fluorescent dextan to LAMP1-mRFP-labeled lysosomes. In these images, it is possible to see the anti-HA signal clearly colocalized with LAMP1 at 5 minutes, but dextran only begins to show only minor colocalization at 15 minues, with better colocalization present only at the 60 minute time point. To quantitiate this data, we determined the percent colocalization of fluorescent (anti-HA or dextran) signal with compartment markers at each time point, and these results were plotted over time (Figure 8B). As expected, we found that βAPP-wt-CFP was internalized to Rab5- compartment within the first 5-10 minutes and only then appears in Rab9-positive compartments at about 15 minutes. However, we observed colocalization of fluorescent HA-antibody labeled βAPP-wt-CFP with LAMP1 at the earliest time points (5, 10 min), when we did not observe detectable colocalization between dextran and LAMP1. βAPP-wt-CFP colocalization with LAMP1 was maximal within 5-10 min of fluorescent HA-antibody labeling, whereas the extent of colocalization with the early (Rab5) and late (Rab9) markers was delayed and was linear over the time period tested, behaving as would be expected for a protein which was first transported to the early endosome, and then to the late endosome. Taken together, the data suggest that while cell surface APP was internalized to the Rab5 compartment, and then transited to the Rab9 compartment as predicted, a large fraction of cell surface APP was rapidly and directly internalized to lysosomes, bypassing endosomal compartments.

### Effect of London and Swedish mutations on the rate of APP trafficking to lysosomes

**Figure 9:**
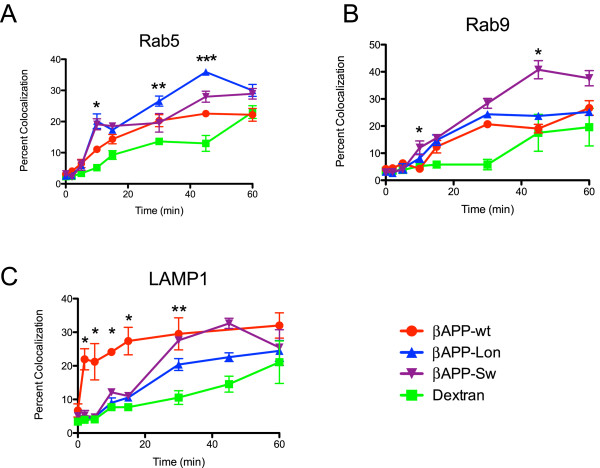
**Internalization of APP mutants into the endosomal/lysosomal system in SN56 cells**. SN56 cells were cotransfected with the indicated βAPP and fluorescent-tagged compartment marker protein constructs as indicated. Cells were then surface labeled with AlexaFluor 647-labeled anti-HA antibody on ice for 30 min and were then allowed to internalize this antibody at 37°C for the time indicated, fixed and imaged. For each time point, the colocalization of the brightest 2% of the anti-HA signal and the compartment marker was quantified (as in Figure 3). **A**. Internalization of surface-labeled APP into the Rab5 compartment (early endosome). * indicates statistically significant difference between the βAPP-SW and βAPP-Lon mutants and all other markers (P < 0.05). ** indicates statistically significant difference between βAPP-Lon, βAPP-wt, and dextran (P < 0.05). *** indicates statistically significant difference between βAPP-Lon, βAPP-SW, and βAPP-wt (P < 0.05). **B**. Internalization of surface-labeled βAPP (wt and mutant) into the Rab9 compartment (late endosome). * indicates statistically significant difference between APP-SW and all other markers. **C**. Internalization of surface-labeled βAPP (wt and mutant) into the LAMP1 compartments. These means were compared statistically by ANOVA at each time point. * indicates statistically significant difference between APP-wt and all other markers (P < 0.05). ** indicates statistically significant difference between βAPP-SW and βAPP-wt from βAPP-Lon and dextran (P < 0.05).

Given the observation that both the London and Swedish mutations altered the extent of APP localization to lysosomes, we compared the time course for mutant and wild-type APP colocalization with early endosomal, late endosomal and lysosomal markers. We found that London and Swedish βAPP exhibited increased colocalization with Rab5 at 10 min. when compared to wild-type βAPP. At 30 minutes, APP-London colocalization with Rab5 was significantly greater that wt-APP and at 45 minutes APP-London colocalization with Rab5 was significantly greater than both APP-Swedish and APP-wt. However, the localization of London, Swedish and wild-type βAPP in the early endosome was indistinguishable at 60 min (Figure 9A). The maximal extent of Swedish βAPP colocalization with Rab9 was substantially increased when compared to either London βAPP or wild-type βAPP suggesting that the Swedish βAPP transited more effectively to late endosomes (Figure 9B). The time course for London βAPP colocalization in late endosomes was virtually indistinguishable from wild-type βAPP. However, unlike what was observed for wild-type βAPP, London and Swedish βAPP did not rapidly colocalize with LAMP1 in lysosomes (Figure 9C) and the appearance of either mutant APP to within this compartment was delayed. While, London βAPP showed reduced transport to the lysosome when compared to wild-type, the transit of Swedish βAPP to the lysosome was accelerated at 30 and 45 min when compared to London βAPP, which was consistent with its increased transport to the late endosomes. Taken together, these data indicated that the kinetics of both London and Swedish βAPP localization to lysosomes was altered when compared with wild-type βAPP. This suggested that distinct intracellular trafficking pathways to the lysosome were being utilized by wild-type and mutant βAPP.

## Discussion

In the present study, we demonstrate that endogenous APP is enriched in neuronal lysosomes as well as lysosomes of Cos7 and SN56 cells that were transfected to overexpress fluorescently tagged APP constructs. In addition to its expected transit from the cell surface to the early endosomal compartment, surface-labeled wild-type APP transits to the lysosomal compartment within minutes and appears to redistribute from the cell surface to LAMP1 vesicles that are found in close proximity to the plasma membrane. Wild-type APP is transported to the lysosome more rapidly than to either early or late endosomes. These observations suggest that APP is trafficked to the lysosome via a mechanism that is distinct from that utilized by the non-specific uptake of dextran. Unexpectedly, two APP mutations that cause increased Aβ peptide accumulation and early onset AD exhibit profound differences in their intracellular trafficking to lysosomes when compared to wild-type APP. Taken together, these experiments suggest distinct trafficking pathways for APP transit from the cell surface to the lysosome.

Although APP has been studied extensively, this is the first demonstration of rapid protein transport directly from the plasma membrane to the lysosome. That APP is highly enriched in lysosomes has been previously observed [28,36-38]. APP has also been demonstrated to move to lysosomes within 15 minutes [7] to 2 hours [10] but these studies did not quantitate either internalization or colocalization. The easy detection of APP in lysosomes suggests that it has a functional role in this compartment, as the half life of lysosomal substrate proteins is 8 minutes and lysosomal substrate proteins are expected to be detectable only at very low levels [39]. One possible parallel might be found in the prion protein (PrP) which normally traffics through early endosomes, but when pathologically misfolded appears to transit to the lysosome without being observed in early endosomal compartments [40,41].

Our data demonstrating enrichment of APP in lysosomes has several limitations. The quantitation method used here is based on colocalization of the brightest pixels. It therefore is likely only useful for comparing colocalization amongst discreet compartments and does not represent an attempt to account for all of the APP in a cell. For example large amounts of APP in other compartments (such as the ER or the plasma membrane) would not be accounted for if they had a lower levels of fluorescence intensity. In addition, compartment marker proteins are often present in other compartments; for example, colocalization with LAMP1 might also include autophagosomes, which have also been identified as a likely site of APP processing [42,43]. This study also specifically ignores possible cleavage events in biosynthetic compartments as β- or γ-cleavage of the construct before transport to the cell surface would remove the HA tag and render these proteins invisible to our cell surface labeling technique.

The appearance of APP in the lysosome before it appears in the early endosomes suggests that wt-βAPP can transit directly to the lysosome from the cell surface, bypassing the early and late endosomal compartments. In contrast both London and Swedish βAPP appear to be excluded from this pathway and transit to the lysosome with kinetics suggesting that they transit first through early endosomes and then through late endosomes. This might account for the fact that we observe less steady-state localization of both London and Swedish APP in lysosomes. Furthermore, the decreased abundance of APP-London in the late endosome, coupled with its accumulation in early endosomes, is consistent with the relative retention of APP-London in the early endosome. Interestingly, Swedish APP is delivered to the late endosome at a faster rate than either wild-type or London mutant APP and yet at steady state there is no apparent difference in the amount of London or wild-type APP in the Rab9 positive late endosome. This suggests that effective proteolysis of APP-Swedish, which is expected to be cleaved by BACE with much higher efficiency than APP-wt [22], may occur as the protein transits from the late endosome to the lysosome. Although this work does not rule out the possibility that some APP might be cleaved by secretases in biosynthetic compartments, it does demonstrate that uncleaved APP (including APP-Swedish) is clearly present on the cell surface. This data also suggests the presence of a novel and highly specific sorting system for APP at the cell surface and a novel pathway from the cell surface directly to the lysosome.

A model to account for the phenomena observed here is that APP might transit to the lysosome by 2 distinct pathways, the classical expected pathway through the early and then late endosomes which has been previously described [7], and another directly from the cell surface to the lysosome either through an intermediate transport vesicle, or by direct fusion and recovery of lysosomes with the plasma membrane. In this model, APP sorting would occur at the cell surface, and APP bearing Swedish and London mutations would be preferentially sorted through the endosomal pathway. There is now good evidence that different cell surface receptors can be directed into distinct clathrin-coated pits based upon binding domains in their intracellular C-terminus [44,45]. This cell surface sorting system has been demonstrated to direct proteins to at least 2 distinct types of endosomes, with some cargo transported to slowly moving, slowly maturing rab5-labeled endosomes, and some cargo sorted to a rapidly maturing Rab7 labeled compartment [46]. Because Rab7 can label both endosomes and lysosomes, this study might also be documenting direct lysosomal transport.

There is no simple explanation for how mutations in the extracellular or transmembrane domain of APP might alter its trafficking. Although proteins involved in APP trafficking typically bind the cytoplasmic C-terminus of APP (reviewed in [47]), there are poorly defined signals in the luminal domain of APP [23,29,48] and deletions of the extracellular juxtamembrane segment of APP have been shown to disrupt APP sorting [49], suggesting that a trafficking mechanism exists which recognizes changes in the extracellular domain of APP. One possible trafficking regulator might be the γ-secretase complex itself (either through presenilin or nicastrin), which can interact with APP in the transmembrane and extracellular domain [50] and only cleaves APP after a β-cleavage has occurred [50,51]. This idea is supported by fact that mutations in PS1 (D385N) that block γ-secretase activity or pharmacological inhibition of γ-secretase activity also block APP internalization [52].

The pathways by which APP transits the endosomal/lysosomal system may have implications for Aβ production. For example, it is possible that the type of proteolysis that APP undergoes may be related to the mechanism by which the protein is being mobilized to the lysosome. Thus, endocytosis through the early and late endosome may lead to more efficient APP cleavage by secretases to produce Aβ while rapid transport to lysosomes might lead to degradation by lysosomal proteases. Because the aggregation of Aβ is also favored by low pH as well as by the intrinsic composition of the lysosomal membrane [53,54], the route of delivery to the lysosome might also affect amyloid formation.

## Conclusions

Although many authors have implicated the lysosome in the pathophysiology of Alzheimer's disease, lysosomes are typically viewed as downstream sites in the trafficking of APP and the production and clearance Aβ. Here we demonstrate that APP is enriched in the lysosome in neuronal cells compared to other endosomal compartments and is rapidly and directly trafficked to the lysosomal compartment. This rapid and specific transport event potentially brings together APP, secretase enzymes, and the conditions, which could either degrade beta amyloid or nucleate its formation into fibrils. This suggests that the endosomal/lysosomal system could play a central role in the pathophysiology of Alzheimer's disease.

## Methods

### Antibodies and Reagents

Cos7 cells were obtained from Dr. Stephen Ferguson. SN56 cells were obtained from Dr. Jane Rylett [30-32]. Serum for cell culture was purchased from Hyclone and cell culture media and reagents were purchased from Invitrogen. Antibodies used in this study were: rabbit anti-APP-C-terminal (Sigma), monoclonal mouse anti-LAMP1 (A3H4) and rat anti-LAMP1 (1D4B) (Developmental Studies Hybridoma Bank), monoclonal anti-Rab5 (BD Biosciences), monoclonal anti-Rab9 (Affinity Bioreagents), and monoclonal anti-HA antibody (12CA5) (Roche Applied Science). Fluorescently-labeled secondary antibodies, AlexaFluor 488 goat anti-rabbit and AlexaFluor 633 donkey anti-mouse were purchased from Invitrogen.

### DNA Constructs

A cDNA encoding APP 750-YFP was a generous gift of Dr. Bradley Hyman. HA-labeled APP constructs were then generated by PCR, first cloning sequence encoding the 17 amino acid signal sequence of APP as well as the L-E residues required for signal peptide cleavage [55] with a forward primer engineered to add an Nhe1 site and a reverse primer encoding the HA sequence and appending an Mlu1 site. The remainder of the cDNA was cloned using PCR primers to place Mlu1 at the 3-prime end and a Sal1 site at the 5-prime end. These 2 products were then ligated into pEYFP-N1 or pECFP-N1 vectors (Clontech). To reduce the possibility of cleavage of APP by a non-secretase enzyme, we generated a shorter constructs using a forward APP primer which would amplify the sequence coding for the C-terminal 112 amino acids (12 amino acids upstream of the β-cleavage site) and append a short 14 amino acid spacer and an Mlu1 site. This construct is referred to as 'βAPP'. Constructs similar to βAPP have been demonstrated to undergo both beta- and gamma-cleavage [56]. We engineered London and Swedish mutations into these constructs using PCR (Figure 1).

LAMP1-YFP was a generous gift from Dr. Walter Mothes and recloned to use mRFP. Cox8-mRFP was a gift from Dr. Mark Huttemann. Plasmids containing mCherry were a gift from Dr. Roger Tsien. Rab9-YFP was obtained from Dr. Susanne Pfeffer and re-cloned to use mCherry.

### Cell Culture and transfection

SN56 cells and COS7 cells were grown in Dulbecco's minimal Eagle's medium (DMEM), respectively supplemented with 5% (v/v) and 10% (v/v) heat-inactivated fetal bovine serum (Hyclone) respectively, and 100 μg/ml penicillin/streptomycin (Invitrogen). Cells were seeded at a density of 2.5 × 10^6 ^cells/100-mm dish (Falcon). Cells were transiently transfected using Lipofectamine following manufacturer's instructions (Invitrogen). Following transfection (18 h), the cells were pooled and reseeded into 35-mm glass-bottomed culture dishes (MatTek) for confocal studies. SN56 cells were grown as above, but were differentiated 24 hours before imaging by the addition of 1 mM dibutyryl cyclic AMP (dbcAMP; Sigma) and changed to serum free medium [30-32].

Primary prefrontal cortical neurons were prepared from E18 mouse embryos as described previously [57], then were seeded in poly-L-ornithine coated plates and maintained in Neurobasal medium supplemented with 1× B27 and 0.8× N2 supplements, 2 mM glutamax and 50 U/ml penicillin/streptomycin (Invitrogen). They were kept at 37°C in a humidified atmosphere containing 5% CO_2_. One third to one half of the volume of neurobasal media was replenished every 3 days. After a growth period of 8-15 days, neurons were processed for immunofluorescence. The University of Western Ontario Animal Care Committee approved all animal protocols.

### Immunocytochemistry

Cells were washed twice in Hanks' balanced salt solution (HBSS; 1.2 mM KH_2_PO_4_, 5 mM NaHCO_3_, 20 mM HEPES, 11 mM glucose, 116 mM NaCl, 4.7 mM KCl, 1.2 mM MgSO_4_, 2.5 mM CaCl_2_, pH 7.4) and fixed for 15 min. in fresh methanol-free 4% paraformaldehyde (Electron Microscopy Supply) in PBS. Cells were permeablized with 0.02% Triton in PBS for 10 min and blocked with 3% BSA in PBS for 1 h. Cells were incubated with primary antibody for 1 hour, washed in PBS twice, incubated with secondary antibody for 1 h. After 2 washes with PBS, cells were stained with Hoechst nuclear stain (Sigma) in PBS for 5 min at room temperature and mounted on slides with ImmuMount (Fisher)

### Confocal Microscopy

Imaging was performed on a Zeiss LSM-510 META laser-scanning microscope using a Zeiss 63× 1.4 numerical aperture oil immersion lens. The optical section thickness was typically 1 micron. AlexaFluor 488, EGFP and YFP fluorescence was visualized using a 488 nm excitation laser and a 500-530-nm emission filter set. AlexaFluor 547 and mRFP fluorescence was imaged using a 543 nm excitation laser and BP 565-615 filter set. ECFP fluorescence was imaged using 458 nm laser excitation source and a BP 475-525 filter set. AlexaFluor 647 fluorescence was imaged using 633 nm excitation laser, and a LP 650 filter. Hoechst signal was collected using a Chameleon Multiphoton laser set at 750 nm excitation, and a 390-465 emission filter set.

### Cell Surface labeling

Anti-HA antibody was labeled with AlexaFluor 647 using a Zenon labeling kit (Invitrogen) following manufacturer's directions. Live cell imaging was performed in HBSS at 37°C on a BC200 microscope stage warmer with a Bionomic BC100 controller from (20/20 Technologies). Cells normal morphology and strong expression of APP and compartment markers were identified for imaging. In live cell imaging experiments, and images were typically taken at 1-2 frames/minute.

For fixed time-course studies, freshly prepared conjugate (10 μg of antibody/ml) was incubated with cells in DMEM on ice for 30 minutes. Conjugate was removed and the cells are washed in cold PBS. Pre-warmed media was then added and cells were incubated at 37°C for the times indicated prior to fixation with 4% paraformaldehyde. Cells were chosen which had strong expression of both the APP and the compartment marker constructs, but had normal morphology and no inclusions. βAPP time courses were performed at least 3 times, with at least 4 representative cells imaged at each time point.

For dextran uptake time courses, AlexaFluor 647-labeled 10 kDa Dextran (Invitrogen) was added to media at 100 μg/ml and cells were incubated for the indicated length of time. Media was then aspirated, cells were washed in ice-cold PBS and fixed 4% paraformaldehyde.

### Colocalization Analysis

Colocalization analysis was performed on confocal optical sections using Imaris 6.1.5 with Imaris Colocalization module (Biplane) running on an Apple Mac Pro to examine the colocalization of the brightest 2% of pixels in each channel. This allows us to set threshold for colocalization in an unbiased manner using the intrinsic properties of the image, eliminating confounding problems caused by variations in cell-to-cell expression and image brightness/exposure thus allowing direct comparison between experiments. Graphing and statistical analysis was performed using Prism GraphPad 5.0b using one-way ANOVA with Tukey post-test.

## Competing interests

The authors declare that they have no competing interests.

## Authors' contributions

AL designed, executed and analyzed many of the internalization assays. Cloned several of the compartment markers and APP constructs. Set up many of the tissue culture conditions. JS carried out and analyzed many of the APP mutant internalization experiments. Subcloned several of the APP constructs. KV developed the initial culture and transfection conditions for SN56 cells. Performed the first sets of transfections with SN56 cells and developed quantitation methods. PHA set up the first culture systems with Cos7 cells. Designed and cloned several of the APP and compartment marker vectors. CS developed and optimized neuronal staining techniques. Cultured and transfected SN56 cells. AM established neuronal culture and immunostaining techniques. SPC developed neuronal culture techniques and provided cultured mouse neurons as well as advice and guidance on neuronal handling and staining. SSGF assisted in design of overall experiments. Provided critical assistance in manpower, input in analysis of data and preparation of the manuscript. SHP is the senior author. Designed overall experiments, analysis and wrote much of the text. Took many of the confocal microscope images. All authors have read and approved the final manuscript.

## Supplementary Material

Additional file 1 **Image stack demonstrating APP preferentially localized in the lysosome a neuronal SN56 cell**. SN56 cells were transiently co-transfected with βAPP-CFP (green) and LAMP1-mRFP (red). Image stack was acquired on a Zeiss LSM510 confocal microscope. The first segment shows βAPP-CFP in green. The second segment of the video shows LAMP1-mrfp overlaid in red. In the third segment, a colocalization channel, showing the colocalization of the brightest 2% of red and green pixels is overlaid in white. QuickTime video was generated using Imaris 6.1.5 software to perform volume rendering and animation. Click here for file

Additional file 2 **Image stack of an immunostained mouse neuron demonstrating APP in the lysosome**. Cultured mouse neurons were fixed and immunolabeled with anti-APP (green) and anti-LAMP1 antibodies (red) and Hoechst nuclear stain (blue). Image stack was acquired on a Zeiss LSM510 confocal microscope. The first segment shows βAPP-CFP in green. The second segment of the video shows LAMP1-mRFP overlaid in red. In the third segment, a channel showing the colocalization of the brightest 2% of red and green pixels is overlaid in white. QuickTime video was generated using Imaris 6.1.5 software to perform volume rendering and animation. Click here for file

Additional file 3 **Movie demonstrating the internalization of FL-APP to the lysosome in a Cos7 cell**. Cos7 cells were transiently transfected with FLAPP-CFP (not shown) and LAMP1-mRFP (red). Fluorescent-labelled anti-HA antibody was added to the media, and a cell was chosen which had good expression of transfected plasmids. Images were acquired using laser-scanning confocal microscopy at approximately 2 frames/min. QuickTime video was generated using Imaris 6.1.5 software. Click here for file

Additional file 4 **Movie demonstrating the internalization of βAPP to the lysosome in a Cos7 cell**. Cos7 cells were transiently transfected with βAPP-CFP (not shown) and LAMP1-mRFP (red). Fluorescent-labelled anti-HA antibody was added to the media, and a cell was chosen which had good expression. Images were acquired using laser-scanning confocal microscopy at approximately 2 frames/min. QuickTime video was generated using Imaris 6.1.5 software. Click here for file
